# Invariant Pattern Recognition with Log-Polar Transform and Dual-Tree Complex Wavelet-Fourier Features

**DOI:** 10.3390/s23083842

**Published:** 2023-04-09

**Authors:** Guangyi Chen, Adam Krzyzak

**Affiliations:** Department of Computer Science and Software Engineering, Concordia University, Montreal, QC H3G 1M8, Canada; krzyzak@cse.concordia.ca

**Keywords:** log-polar transform, dual-tree complex wavelet transform (DTCWT), fast Fourier transform (FFT), discrete wavelet transform (DWT), pattern recognition

## Abstract

In this paper, we propose a novel method for 2D pattern recognition by extracting features with the log-polar transform, the dual-tree complex wavelet transform (DTCWT), and the 2D fast Fourier transform (FFT2). Our new method is invariant to translation, rotation, and scaling of the input 2D pattern images in a multiresolution way, which is very important for invariant pattern recognition. We know that very low-resolution sub-bands lose important features in the pattern images, and very high-resolution sub-bands contain significant amounts of noise. Therefore, intermediate-resolution sub-bands are good for invariant pattern recognition. Experiments on one printed Chinese character dataset and one 2D aircraft dataset show that our new method is better than two existing methods for a combination of rotation angles, scaling factors, and different noise levels in the input pattern images in most testing cases.

## 1. Introduction

Pattern recognition is a very important topic in computer vision. It is extremely useful in optical character recognition (OCR), face recognition, iris recognition, fingerprint recognition, palmprint recognition, etc. Furthermore, feature extraction from 2D pattern images is a crucial step in invariant pattern recognition [1]. Most existing methods lack the invariant property, which is undesirable in real-life applications. For example, translation invariance, rotation invariance, and scaling invariance are very important in invariant pattern recognition. Pattern recognition can automatically recognize patterns in data, which can be anything from text and images to sounds or other definable qualities. It can recognize 2D pattern images quickly and precisely.

In this paper, we propose to extract invariant features by the log-polar transform [2], the dual-tree complex transform (DTCWT [3]), and the 2D fast Fourier transform (FFT2 [4]). The DTCWT transform decomposes the pattern image in a multiresolution way, and it is invariant to spatial shift, which is very important in pattern recognition. We know that very low-resolution sub-bands lose fine features in the pattern images, and very high-resolution sub-bands contain a significant amount of noise [5]. Hence, intermediate-resolution sub-bands are extremely good for invariant pattern recognition. Our extracted features are invariant to translation, rotation, and scaling. Experiments show that our new method is better than the log-polar-FFT2 method, and the log-polar discrete wavelet transform (DWT [6])-FFT2 method for recognizing printed Chinese characters and 2D aircraft in most testing cases. This demonstrates that our new method is very useful in many real-life applications.

The organization of this paper is as follows. Section 2 proposes a novel method for invariant pattern recognition. Section 3 describes an experiment conducted to test the effectiveness of our proposed method. Finally, Section 4 draws the conclusion of the paper and introduces future research directions.

## 2. Proposed Method

The log-polar transform converts rotation and scaling into spatial shifts. Mathematically speaking, it is a coordinate system in two dimensions, where a point is identified by two numbers, one for the logarithm of the distance to a certain point, and one for an angle. Let image F_2_ be the translated, rotated, and scaled version of image F_1_; then they are correlated as

$$F_{2}(r,\theta )=F_{1}(\frac{r}{a},\theta -\theta_{0})$$

$$F_{2}(\log r,\theta )=F_{1}(\log r-\log a,\theta -\theta_{0})$$

$$F_{2}(\xi ,\theta )=F_{1}(\xi -d,\theta -\theta_{0})$$

where

ξ=log⁡r

d=log⁡a.

As a result, their Fourier spectra will be invariant to translation, rotation, and scaling of the input pattern images. This is because the magnitudes of the Fourier coefficients are invariant to spatial shifts.

The DTCWT can decompose the image into multiresolution scales in a translation- invariant way. It computes the complex transform of an image by using two separate DWT decompositions, namely, tree a and tree b. If the filters used in one are specifically designed differently from those in the other, it is possible for one DWT to produce the real coefficients and the other the imaginary. Taking the FFT2 transform of each DTCWT sub-band coefficient will result in translation-invariant sub-band coefficients, so that we can recognize each pattern image effectively. In this paper, we use the 3rd, 4th, and 5th sub-band coefficients of the DTCWT transform for the recognition of printed Chinese characters and 2D aircraft. It is clear thar very low-resolution sub-bands lose important features in the pattern images and very high-resolution sub-bands contain a lot of noise. As a result, intermediate-resolution sub-bands are desirable for invariant pattern recognition.

Our newly proposed method in this paper can be summarized as follows:

➢ Translate the input pattern image to the centroid of the pattern.
➢ Convert the pattern from cartesian coordinates to log-polar coordinates of 128 × 128 pixels in size. Let us denote it as LP.
➢ Perform DTCWT transform on the log-polar image LP for K = 5 decomposition scales.
➢ Construct complex sub-bands: COMPLEX (k) = Tree a (k) + i × Tree b (k), kϵ[1,K].
➢ Conduct FFT2 transform for each COMPLEX sub-band, and take their spectra.
➢ Recognize the pattern image to one known class by using the 3rd, 4th, and 5th sub-bands of the computed transform coefficients.
➢ Compute the correct recognition rate for the testing dataset by using the nearest neighbor (NN) classifier.

The FFT2 [3] converts an image from its original domain in space to a representation in the frequency domain. It can reduce the complexity of computing the discrete Fourier transform (DFT) of an image from O((M × N)^2^) to O(M × N log (M × N)), where M and N are the row and column numbers of the pattern image. The Fourier spectra are invariant to spatial shifts, which is very important for invariant pattern recognition.

We also compare our new method with two existing methods: log-polar FFT2 and log-polar DWT-FFT2. The first method computes the log-polar transform and then takes the FFT2 to obtain invariant features. The second method performs the log-polar transform, the DWT transform [4], and the FFT2 transform to obtain invariant features.

Our invariant pattern recognition method is still useful and competitive when compared to convolutional neural networks (CNN). It can extract invariant features from pattern images quickly, instead of training a CNN for many hours. Our new method achieves very high classification accuracies for two datasets and for different combinations of deformations in the input pattern images, as demonstrated in the experimental section in the paper.

The computational complexity of this paper can be given as follows. Let the pattern image be of size M × N. The log-polar transform is a linear operation with complexity O(M × N). The DTCWT transform is a linear operation with complexity O(M × N). The FFT2 is in the complexity of O(M × N log(M × N)). As a result, the total complexity of our new method is O(M × N log(M × N)).

The major contribution of this paper is that we have successfully extracted very stable features in multiresolution from the pattern images, which are invariant to translation, rotation, and scaling. It is not common to see published papers that achieve all three invariant properties in a multiresolution way. It is well-known that very low-resolution sub-bands lose important features in the pattern images, and very high-resolution sub-bands contain a lot of noise. Consequently, intermediate-resolution sub-bands are very good for invariant pattern recognition. Experimental results demonstrate that our new method proposed in this paper is better than the log-polar FFT2 method and the log-polar DWT-FFT2 method in most testing cases for recognizing printed Chinese characters and 2D aircraft.

## 3. Experiments

**Figure 1:**
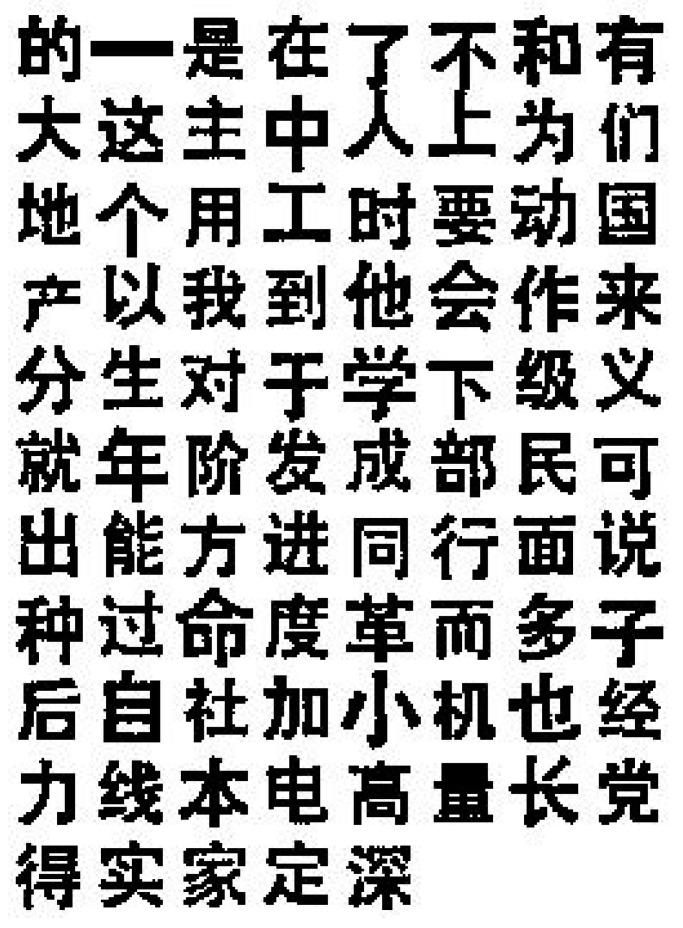
The printed Chinese character dataset.

**Figure 2:**
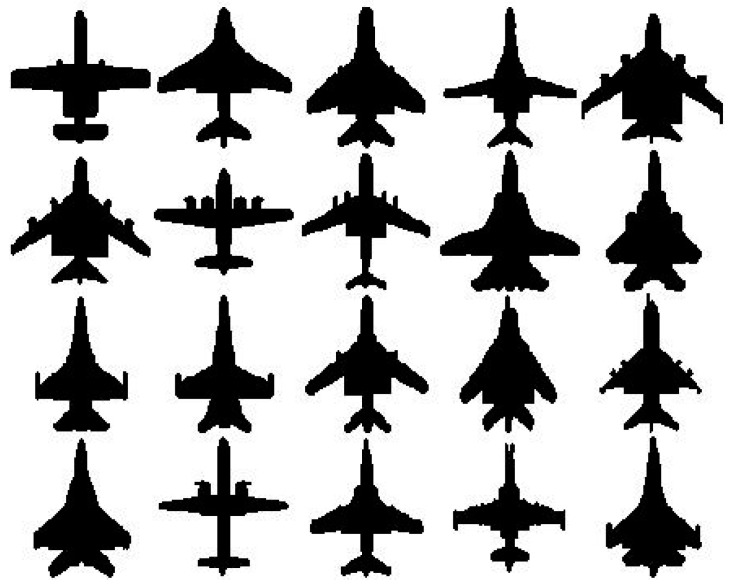
The 2D aircraft dataset.

**Figure 3:**
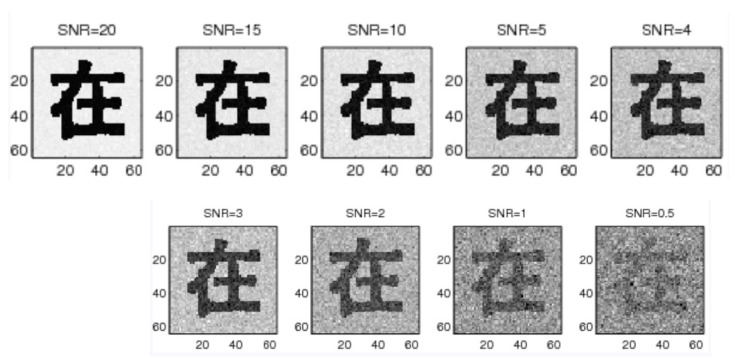
The noisy images with SNR = 20, 15, 10, 5, 4, 3, 2, 1, and 0.5, respectively.

**Figure 4:**
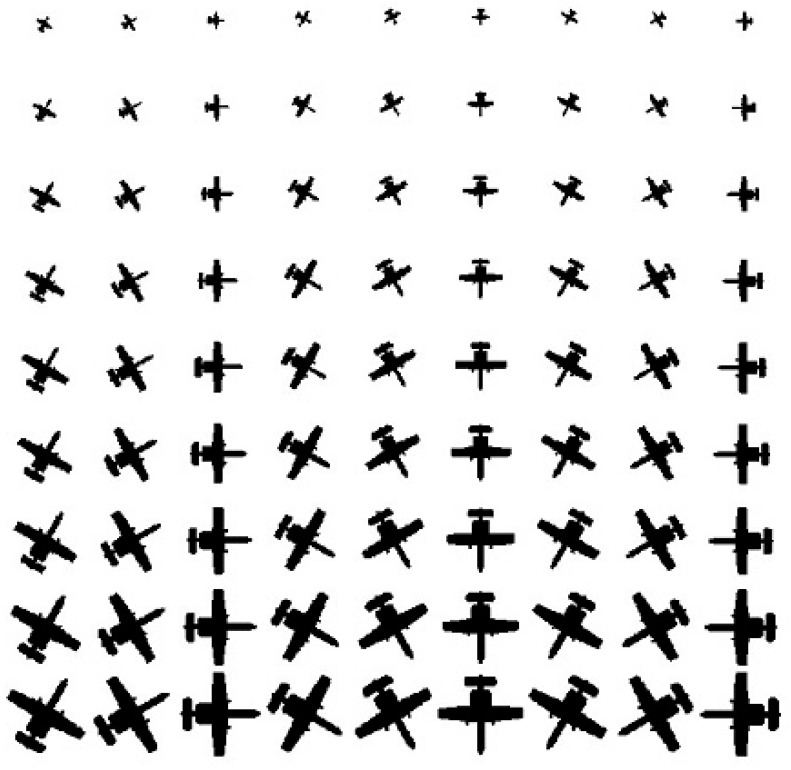
A combination of scaling factors (0.2, 0.3, 0.4, 0.5, 0.6, 0.7, 0.8, 0.9, and 1.0) and rotation angles (30°, 60°, 90°, 120°, 150°, 180°, 210°, 240°, and 270°) for an aircraft image.

We conducted experiments with one printed Chinese character dataset (Figure 1) and one 2D aircraft dataset (Figure 2), where 85 characters and 20 aircraft exist in each dataset, respectively. Both datasets are in binary format. We performed experiments with the proposed method in this paper, the log-polar-FFT2 method, and the log-polar DWT-FFT2 method. We deformed the input pattern images with scaling factors 0.2, 0.3, 0.4, 0.5, 0.6, 0.7, 0.8, 0.9, and 1.0. We rotated the input pattern images with 30°, 60°, 90°, 120°, 150°, 180°, 210°, 240° and 270°. We also added noise to the input pattern images with signal-to-noise-ratio (SNR) = 20, 15, 10, 5, 4, 3, 2, 1 and 0.5 (Figure 3). The SNR is defined as:

$$SNR=\frac{\sqrt{\sum {\left(F\left(i,j\right)-avg(F)\right)}^{2}}}{\sqrt{\sum {\left(n\left(i,j\right)-avg(n)\right)}^{2}}}$$

where *F* is the noise-free image, *n* is the added Gaussian white noise, and *avg*(*F*) and *avg*(*n*) are the average values of the image *F* and image *n*, respectively. A combination of rotation angles and scaling factors for an aircraft is shown in Figure 4.

Our experimental results are demonstrated as follows. Table 1 tabulates the correct recognition rates for a combination of rotation angles and scaling factors for the proposed method for the printed Chinese character dataset. Table 2 displays the correct recognition rates for a combination of rotation angles and scaling factors for the log-polar-FFT2 method for the printed Chinese character dataset. Table 3 shows the correct recognition rates for a combination of rotation angles and scaling factors for the log-polar DWT-FFT2 method for the printed Chinese character dataset. Table 4 tabulates the correct recognition rates for a combination of rotation angles and scaling factors for the proposed method for the 2D aircraft dataset. Table 5 shows the correct recognition rates for a combination of rotation angles and scaling factors for the log-polar-FFT2 method for the 2D aircraft dataset. Table 6 shows the correct recognition rates for a combination of rotation angles and scaling factors for the log-polar DWT-FFT2 method for the 2D aircraft dataset. Table 7 tabulates the correct recognition rates for a combination of rotation angles and noise levels for the proposed method for the printed Chinese character dataset. Table 8 shows the correct recognition rates for a combination of rotation angles and noise levels for the log-polar-FFT2 method for the printed Chinese character dataset. Table 9 lists the correct recognition rates for a combination of rotation angles and noise levels for the log-polar DWT-FFT2 method for the printed Chinese character dataset. Table 10 shows the correct recognition rates for a combination of rotation angles and noise levels for the proposed method for the 2D aircraft dataset. Table 11 tabulates the correct recognition rates for a combination of rotation angles and noise levels for the log-polar-FFT2 method for the 2D aircraft dataset. Table 12 shows the correct recognition rates for a combination of rotation angles and noise levels for the log-polar DWT-FFT2 method for the 2D aircraft dataset.

From Table 1, Table 2, Table 3, Table 4, Table 5, Table 6, Table 7, Table 8, Table 9, Table 10, Table 11 and Table 12, we can see that our proposed method in this paper performs the best in most testing cases. Our new method is better than both the log-polar-FFT2 method and the log-polar DWT-FFT2 method for a combination of rotation and scaling factors and different noise levels in most testing cases. Our new method is not as good as existing methods in rare cases in our experiments. Furthermore, our new method is fast as well, in terms of CPU computational time for invariant pattern recognition.

## 4. Conclusions

Invariant pattern recognition is an extremely important topic in today’s computer vision applications. For example, it is very useful in OCR and biometrics such as face recognition, iris recognition, palmprint recognition, fingerprint recognition, and so forth. Furthermore, extracting invariant features from 2D pattern images is very useful for many real-life applications.

In this paper, we have proposed a novel method for pattern recognition by using the log-polar transform, the DTCWT transform, and the FFT2 transform. Our extracted features are invariant to translation, rotation, and scaling in a multiresolution way. It is well-known that very low-resolution sub-bands lose fine features in the pattern images, and very high-resolution sub-bands contain significant amounts of noise. Hence, intermediate-resolution sub-bands are very good for invariant pattern recognition. Experiments demonstrate that our new method is better than the log-polar-FFT2 method and the log-polar DWT-FFT2 method for recognizing printed Chinese characters and 2D aircraft in most testing cases.

Future research will be conducted by introducing denoising to the pattern images so that better recognition results can be obtained. For instance, we can use our previously published image denoising methods [7,8,9,10] to preprocess the input pattern images. We will also study deep convolutional neural networks (DNN) for invariant pattern recognition, which have achieved amazing results in recent years in real-life applications.

## Figures and Tables

**Table 1:** The correct recognition rates for a combination of rotation angles and scaling factors for the proposed method for the printed Chinese character dataset.

Scaling Factor	Rotation								
	30°	60°	90°	120°	150°	180°	210°	240°	270°
0.2	7.06	10.59	9.41	7.06	11.76	9.41	7.06	11.76	9.41
0.3	27.06	23.93	50.59	24.71	22.35	42.35	23.53	22.35	40.00
0.4	57.65	60.00	88.24	57.65	60.00	88.24	57.65	60.00	88.24
0.5	76.47	84.71	88.24	80.00	81.18	94.12	83.53	82.35	95.29
0.6	97.65	96.47	97.65	97.65	96.47	97.65	97.65	96.47	97.65
0.7	100	100	100	100	98.82	100	100	98.82	100
0.8	100	100	100	100	100	100	100	100	100
0.9	100	100	100	100	100	100	100	100	100
1.0	100	100	100	100	100	100	100	100	100

**Table 2:** The correct recognition rates for a combination of rotation angles and scaling factors for the log-polar-FFT2 method for the printed Chinese character dataset.

Scaling Factor	Rotation								
	30°	60°	90°	120°	150°	180°	210°	240°	270°
0.2	5.88	5.88	8.24	5.88	5.88	8.24	7.06	7.09	8.24
0.3	24.71	21.18	35.29	25.88	18.82	30.59	25.88	20.00	27.06
0.4	43.53	47.06	64.71	43.53	48.24	64.71	43.53	47.06	64.71
0.5	64.71	71.76	76.47	72.94	75.29	76.47	70.59	67.06	78.82
0.6	97.65	98.82	95.29	97.65	98.82	95.29	97.65	98.82	95.29
0.7	100	100	100	100	100	100	100	100	100
0.8	100	100	100	100	100	100	100	100	100
0.9	100	100	100	100	100	100	100	100	100
1.0	100	100	100	100	100	100	100	100	100

**Table 3:** The correct recognition rates for a combination of rotation angles and scaling factors for the log-polar DWT-FFT2 method for the printed Chinese character dataset.

Scaling Factor	Rotation								
	30°	60°	90°	120°	150°	180°	210°	240°	270°
0.2	4.71	9.41	5.88	4.71	9.41	5.88	4.71	9.41	5.88
0.3	21.18	18.82	48.24	20.00	20.00	45.88	20.00	20.00	37.65
0.4	40.00	44.71	84.71	40.00	44.71	84.71	40.00	44.71	84.71
0.5	58.82	70.59	88.24	69.41	62.35	92.94	62.35	55.29	95.29
0.6	85.88	88.24	98.82	87.06	87.06	98.82	85.88	87.06	98.82
0.7	96.47	94.12	98.82	94.12	92.94	98.82	96.47	91.76	100
0.8	98.82	95.29	100	98.82	95.29	100	98.82	95.29	100
0.9	98.82	100	100	98.82	100	100	100	100	100
1.0	100	100	100	100	100	100	100	100	100

**Table 4:** The correct recognition rates for a combination of rotation angles and scaling factors for the proposed method for the 2D aircraft dataset.

Scaling Factor	Rotation								
	30°	60°	90°	120°	150°	180°	210°	240°	270°
0.2	65	55	70	65	55	70	65	55	70
0.3	90	95	100	90	95	95	95	90	100
0.4	100	100	100	100	100	100	100	100	100
0.5	100	100	100	100	100	100	100	100	100
0.6	100	100	100	100	100	100	100	100	100
0.7	100	100	100	100	100	100	100	100	100
0.8	100	100	100	100	100	100	100	100	100
0.9	100	100	100	100	100	100	100	100	100
1.0	100	100	100	100	100	100	100	100	100

**Table 5:** The correct recognition rates for a combination of rotation angles and scaling factors for the log-polar-FFT2 method for the 2D aircraft dataset.

Scaling Factor	Rotation								
	30°	60°	90°	120°	150°	180°	210°	240°	270°
0.2	65	60	65	65	60	65	65	60	65
0.3	95	100	100	100	100	95	100	100	100
0.4	100	100	100	100	100	100	100	100	100
0.5	100	100	100	100	100	100	100	100	100
0.6	100	100	100	100	100	100	100	100	100
0.7	100	100	100	100	100	100	100	100	100
0.8	100	100	100	100	100	100	100	100	100
0.9	100	100	100	100	100	100	100	100	100
1.0	100	100	100	100	100	100	100	100	100

**Table 6:** The correct recognition rates for a combination of rotation angles and scaling factors for the log-polar DWT-FFT2 method for the 2D aircraft dataset.

Scaling Factor	Rotation								
	30°	60°	90°	120°	150°	180°	210°	240°	270°
0.2	30	30	60	30	30	60	30	30	60
0.3	80	75	100	60	75	95	70	75	100
0.4	90	95	100	90	95	100	90	95	100
0.5	95	100	100	95	95	100	100	95	100
0.6	100	100	100	100	100	100	100	100	100
0.7	100	100	100	100	100	100	100	100	100
0.8	100	100	100	100	100	100	100	100	100
0.9	100	100	100	100	100	100	100	100	100
1.0	100	100	100	100	100	100	100	100	100

**Table 7:** The correct recognition rates for a combination of rotation angles and noise levels for the proposed method for the printed Chinese character dataset.

SNR	Rotation								
	30°	60°	90°	120°	150°	180°	210°	240°	270°
20	100	100	100	100	100	100	100	100	100
15	100	100	100	100	100	100	100	100	100
10	100	100	100	100	100	100	100	100	100
5	100	100	100	100	100	100	100	100	100
4	100	100	100	100	100	100	100	100	100
3	100	100	100	100	100	100	100	100	100
2	100	100	100	100	100	100	100	100	100
1	96.47	97.65	100	94.12	96.47	98.82	98.82	94.12	98.82
0.5	14.12	17.65	34.12	10.59	14.12	34.12	20.00	10.59	40.00

**Table 8:** The correct recognition rates for a combination of rotation angles and noise levels for the log-polar-FFT2 method for the printed Chinese character dataset.

SNR	Rotation								
	30°	60°	90°	120°	150°	180°	210°	240°	270°
20	100	100	100	100	100	100	100	100	100
15	100	100	100	100	100	100	100	100	100
10	100	100	100	100	100	100	100	100	100
5	100	100	100	100	100	100	100	100	100
4	100	100	100	100	100	100	100	100	100
3	100	100	100	100	100	100	100	100	100
2	100	100	100	100	100	100	100	100	100
1	84.71	80.00	91.76	83.53	81.18	92.94	84.71	75.29	95.29
0.5	7.06	4.71	9.41	1.18	4.71	7.06	4.71	4.71	10.59

**Table 9:** The correct recognition rates for a combination of rotation angles and noise levels for the log-polar DWT-FFT2 method for the printed Chinese character dataset.

SNR	Rotation								
	30°	60°	90°	120°	150°	180°	210°	240°	270°
20	100	100	100	100	100	100	100	100	100
15	100	100	100	100	100	100	100	100	100
10	100	100	100	100	100	100	100	100	100
5	100	100	100	100	100	100	100	100	100
4	100	100	100	98.82	100	100	100	100	100
3	100	100	100	100	100	100	98.82	100	100
2	98.82	100	100	98.82	100	100	97.65	100	100
1	84.71	84.71	100	82.35	87.06	100	82.35	85.88	100
0.5	11.76	11.76	32.94	11.76	10.59	40.00	7.06	12.94	32.94

**Table 10:** The correct recognition rates for a combination of rotation angles and noise levels for the proposed method for the 2D aircraft dataset.

SNR	Rotation								
	30°	60°	90°	120°	150°	180°	210°	240°	270°
20	100	100	100	100	100	100	100	100	100
15	100	100	100	100	100	100	100	100	100
10	100	100	100	100	100	100	100	100	100
5	100	100	100	100	100	100	100	100	100
4	100	100	100	100	100	100	100	100	100
3	100	100	100	100	100	100	100	100	100
2	100	100	100	100	100	100	100	100	100
1	90	95	100	95	100	100	80	90	100
0.5	15	10	10	15	10	20	5	10	20

**Table 11:** The correct recognition rates for a combination of rotation angles and noise levels for the log-polar-FFT2 method for the 2D aircraft dataset.

SNR	Rotation								
	30°	60°	90°	120°	150°	180°	210°	240°	270°
20	100	100	100	100	100	100	100	100	100
15	100	100	100	100	100	100	100	100	100
10	100	100	100	100	100	100	100	100	100
5	100	100	100	100	100	100	100	100	100
4	100	100	100	100	100	100	100	100	100
3	100	100	100	100	100	100	100	100	100
2	100	100	100	100	100	100	100	100	100
1	70	65	60	55	45	45	50	50	70
0.5	15	15	15	15	10	10	15	15	15

**Table 12:** The correct recognition rates for a combination of rotation angles and noise levels for the log-polar DWT-FFT2 method for the 2D aircraft dataset.

SNR	Rotation								
	30°	60°	90°	120°	150°	180°	210°	240°	270°
20	100	100	100	100	100	100	100	100	100
15	100	100	100	100	100	100	100	100	100
10	100	100	100	100	100	100	100	100	100
5	100	100	100	100	100	100	100	100	100
4	100	100	100	100	100	100	100	100	100
3	100	100	100	100	100	100	100	100	100
2	100	100	100	95	100	100	95	100	100
1	60	80	100	55	75	95	65	80	100
0.5	10	5	20	5	10	25	5	5	20

## Data Availability

Data sharing not applicable to this article as no datasets were generated or analyzed during the current study.
